# May New Locally Acquired Cases of COVID-19 Have Been Linked to Illegal Entry into Vietnam?

**DOI:** 10.1017/dmp.2020.414

**Published:** 2020-10-26

**Authors:** Trang H. D. Nguyen

**Affiliations:** Institute of Biotechnology and Food Technology, Industrial University of Ho Chi Minh City, Ho Chi Minh City, Vietnam

**Keywords:** COVID-19, illegal entry, local transmission, Vietnam

Vietnam imposed a stay-at-home order and 15 days of nationwide social distancing from April 1, 2020, to combat community transmission of the new coronavirus. As of April 1, the country documented 212 positive cases of coronavirus disease (COVID-19), with 63 of these having recovered.^1^ On April 23, the Government of Vietnam eased social distancing measures for almost all localities across the country after seeing no daily new cases within its borders. This facilitated repatriation of thousands of stranded overseas Vietnamese nationals and resumption of domestic trade activities. To prevent local transmission of the new coronavirus in the community after the lifting of social distancing guidelines, Vietnam mandates health checks and 14-day quarantine at government-run facilities for those arriving in the country; and, though the government has made tremendous effort in controlling and monitoring closely repatriation, immigration, and foreign entry, it remains a concern about the potential spread of COVID-19 due to those illegally entering Vietnam borders.

From July 24 to 27, 2020, the country reported 15 new confirmed local infections of COVID-19 in Danang, a city in the Central Vietnam, ending 99 days without any case of community transmission. The occurrence raises a question as to whether these new local cases are attributed to illegal entry into Vietnam. As of September 12, Vietnam has confirmed 1063 positive cases of COVID-19 with 35 deaths. Currently, 35 799 COVID-19 suspected cases are under quarantine in centralized facilities, hospitals, and self-quarantine at home.

As part of a precaution approach to preventing and controlling the second wave of COVID-19, the Government of Vietnam has imposed reinforced border control measures and 14-day quarantine on arrival. The logic for this border control in a tight matter comes from the well-known knowledge that travels can facilitate rapid dissemination of the pandemic.^2^ The advantage of this strategy is to discourage a massive illegal entry from China, Laos, and Cambodia, which share long borders with Vietnam, and thus effectively contain the potential spread of COVID-19 due to asymptomatic COVID-19 patients illegally crossing the borders. However, increases in the number of border authorities, including local authorities who ransack and monitor unlawful residents, and security check points, may lead to financial burdens on the country. In contrary to border control, controlling the population within the national territory is likely to overload the health care system of Vietnam, which has lacked preparedness for a second wave of devastating, unforeseeable outbreak. This unreinforced border control could encourage unlawful entries from neighboring countries as well. Nevertheless, the use of this option would lessen a financial burden of the government.

Vietnam shares long land borders and has a high frequency and variety of trans-border movements of consumer goods, capital, and people in the legal economy with its neighboring countries (ie, Cambodia, China, and Laos). Although border permeability has increased in the past few decades, law enforcement agencies have failed to improve their capacity to filter cross-border movements efficiently.^3^ Particularly during this COVID-19 pandemic in Vietnam, many repatriates have made attempts to enter and exit the country through unmonitored trails along its borders. Perhaps this is to evade 14-day centralized quarantine, which is often thought to lead to neighborhood stigma. Recently, a man who illegally returned to Vietnam from Cambodia has been confirmed infected with the new coronavirus,^4^ and, though this patient is now quarantined and treated at a provincial hospital, concerns have increased over the spread of the new coronavirus due to his previous close contact with healthy people. As both Cambodia and Vietnam confirm that no new locally acquired infections have been recorded over the past 30 days within their borders, it is difficult to trace the source of this COVID-19 case. In addition to the evasion of quarantine, it is noted that, during this unprecedented pandemic, fear and anxiety about the disease could incite people to flee their hard-hit countries and illegally cross land borders. For example, in early March, when China was still at its peak of COVID-19, 4 Chinese citizens illegally entered the border into North Vietnam to escape the new coronavirus. Since mid-July, 45 foreigners, mostly Chinese nationals, were found to illegally enter or unlawfully reside in Danang, which is currently on re-lockdown due to the new local transmissions.^4^ Though those people are now quarantined and tested negative for severe acute respiratory syndrome coronavirus 2 (SARS-CoV-2), such illegal entries hint at a potential threat of a second wave of COVID-19 in the country. This would definitely damage socioeconomic activities and hinder the fight against COVID-19. The fear about spread of the disease increased when on August 12, a Chinese man who unlawfully crossed the border tested positive for SARS-CoV-2.^5^ The country, so far, has recorded more than 80 illegal entries, including 18 Vietnamese repatriates, at least 49 Chinese, and 5 Laotian citizens.^5^ Previously, we reported that the majority (60%) of SARS-CoV-2 infections in Vietnam were acquired overseas.^1^ The figure has been increasing due to the infected repatriates since the easing of travel restrictions.

The country has released a document with detailing penalties for violations of COVID-19 prevention regulations. Thereby, an administrative fine of VN $5–10 million (US $215–430) would be imposed on those evading mandatory quarantine. Failure to report health status fully and accurately would result in criminal charges. Entry into the country without performing immigration procedures is punishable by fines of up to VN $3–5 million (US $130–215) or criminal charges.

Additional measures should be implemented to prevent a potential spread of COVID-19 due to illegal entry:

1. Checkpoints need to be set up on trails and roads along the land borders between Vietnam and the neighboring countries. A security check and registration of cross-border travelers and vehicles need to be strengthened.
2. Local authorities need to work in coordination with relatives and neighbors of illegal returnees.
3. Vietnamese border authorities should work in coordination with their counterparts from the neighboring countries.
4. Trained dogs should be used to prevent illegal entry.

In summary, illegal entry into Vietnam via land borders amid the COVID-19 pandemic deserves greater attention, as the evolution of the pandemic is complicated and hardly predictable. It could cause local transmission of the new coronavirus, negatively affecting containment efforts of the country. Therefore, the country needs to impose strict preventive and control measures to closely monitor repatriation and immigration via its land borders.
